# Common bile duct stones: an unusual case of diarrhoea through a mucous fistula

**DOI:** 10.1308/003588412X13373405384936

**Published:** 2012-10

**Authors:** PT Davey, E Epanomeritakis, A Moorehead

**Affiliations:** Southern Health and Social Care Trust,UK

**Keywords:** Gallstones, Cholecystocolonic fistula, Diarrhoea

## Abstract

We present a very unusual case of diarrhoea in a 77-year-old man. He had a previously complicated surgical history, with a loop ileostomy and a colonic mucous fistula. He developed a sudden onset of diarrhoea from his mucous fistula. A contrast enema suggested a cholecystocolonic fistula and subsequent computed tomography demonstrated a common bile duct stone caused a degree of obstruction. The patient was treated successfully by endoscopic retrograde cholangiopancreatography and stone extraction. This case demonstrated the role that contrast enema may still play in unusual cases of diarrhoea.

## Case history

A 77-year-old man presented through the emergency department. He had a background history of a prostatic carcinoma. He presented with severe right iliac fossa pain and iron deficit anaemia. Urgent computed tomography (CT) of the abdomen and pelvis demonstrated perforated diverticular disease in the sigmoid colon. He proceeded to an emergency laparotomy and underwent a Hartmann’s procedure.

The patient deteriorated acutely on day 31 following his initial surgery. CT suggested a rectal stump distended with fluid and multiple air/fluid collections. This was managed initially with percutaneous radiological drainage. On day 37, he developed signs in keeping with adhesional small bowel obstruction, confirmed with CT. Conservative management failed and he proceeded to a further laparotomy. The rectal stump had broken down, precipitating a pelvic abscess with adhesive small bowel obstruction. The surgeon fashioned a loop ileostomy in the right iliac fossa following a small bowel resection.

The patient re-presented to the outpatient setting 14 months later. He had been managing quite well with the loop ileostomy and the mucous fistula but complained that there was a sudden increase in the volume of output of the mucous fistula. An enterocolonic fistula was suspected and a water soluble contrast enema was organised. On insufflation with contrast and air, the radiologist noted an abnormality close to the hepatic flexure. Further imaging demonstrated the presence of a cholecystocolonic fistula and the contrast study demonstrated the biliary anatomy.

CT was requested to assess this region further. This demonstrated both air and stones in the gallbladder. The common bile duct was distended with a stone noted at the lower end (Fig 3). Review of the previous CT also demonstrated air and calculi in the gallbladder with a normal common bile duct, suggesting the fistula was present prior to the laparotomies.

**Figure 1:**
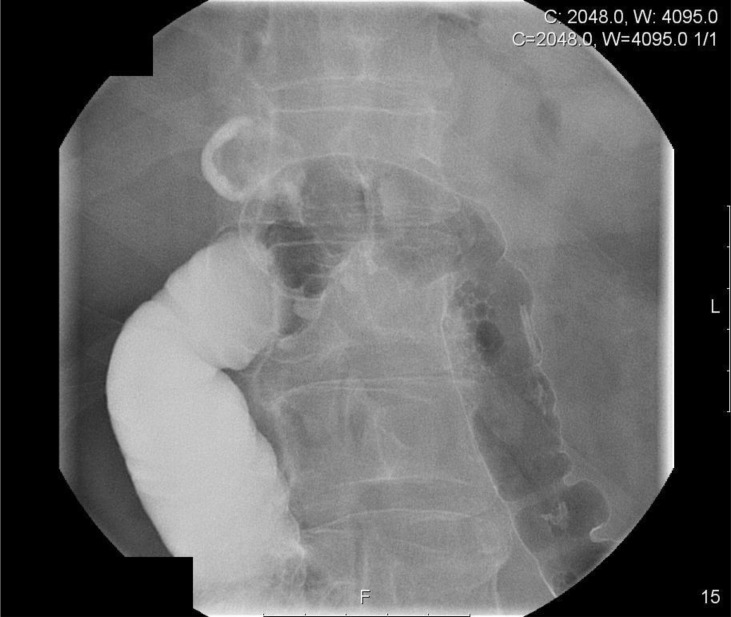
Contrast enema study demonstrating abnormality at the hepatic flexure

**Figure 2:**
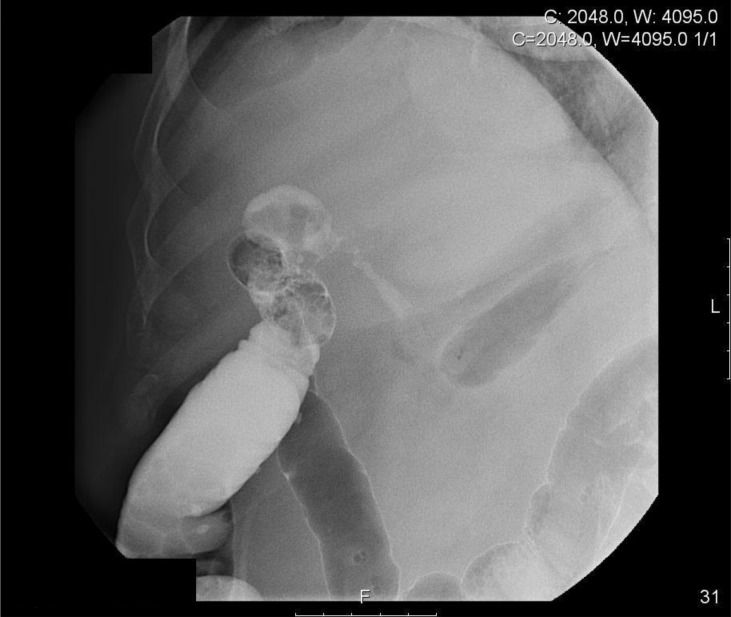
Contrast enema study demonstrating contrast in the gallbladder, cystic duct and common bile duct

**Figure 3:**
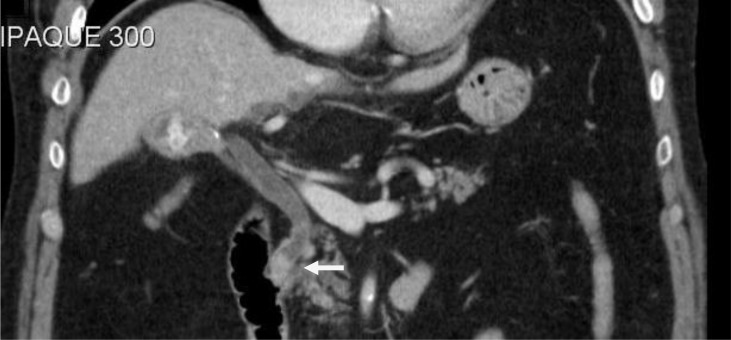
Coronal computed tomography demonstrating a stone in the gallbladder and a stone at the lower end of a distended common bile duct

It was felt that the sudden onset of diarrhoea from this patient’s mucous fistula was precipitated by the passage of a gallstone in the common bile duct, causing a degree of biliary obstruction that discharged through the cholecystocolonic fistula. He underwent endoscopic retrograde cholangiopancreatography (ERCP) and stone extraction. In view of his extensive co-morbidities, routine cholecystectomy was not planned.

## Discussion

Cholecystocolonic fistula is a rare entity encountered by a general surgeon, occurring in 0.13% of cases of calculus cholecystitis^1^ with a prevalence of 0.22% at autopsy.^2^ It is associated primarily with chronic cholecystitis. Occasionally, it can occur in the presence of complicated diverticular disease, Crohn’s disease, colonic adenocarcinoma and abdominal trauma, and in approximately 2% of cases it can be associated with gallbladder carcinoma.^3^ Cholecystocolonic fistulas affect women more commonly and their incidence is maximal in the sixth and seventh decades.

The presentation usually involves chronic diarrhoea. One report describes a cholecystocolonic fistula eventually communicating to the skin.^4^ Cholecystocolonic fistulas have a low pre-operative pick up rate, more commonly being found intra-operatively.

Investigations for chronic diarrhoea generally involve a colonoscopy that has been reported to have detected a cholecystocolonic fistula previously.^5^ Other modalities include CT, which may demonstrate pneumobilia and pericholecystic inflammatory changes (as in our case), ERCP and contrast enema studies.

Management can follow many routes depending on the fitness of the patient. Operative intervention can include cholecystectomy with or without a segmental resection of the colon. Non-operative management includes ERCP with sphincterotomy and drainage to minimise the volume of bile through the fistula.

## Conclusions

Our case has demonstrated that there may still be a role for performing a contrast enema in a patient with diarrhoeal symptoms and a history of gallstone pathology even if the bowel endoscopy was normal.
